# Supplementary material for: Characteristics and Outcomes of Stem Cell Transplant Patients during the COVID-19 Era: A Systematic Review and Meta-Analysis
Source: Healthcare (Basel). 2024 Feb 23;12(5):530. doi: 10.3390/healthcare12050530 (PMC10931059; doi:10.3390/healthcare12050530)
Supplement: Supplementary file 1 [file healthcare-12-00530-s001.zip › healthcare-2856189-supplementary.pdf]

Supplementary Table S1. MEDLINE search strategy.

Search number	Search terms
1	COVID-19.rx,px,ox. or severe acute respiratory syndrome coronavirus 2.os.
2	("COVID-19" or COVID19 or "COVID 2019" or "novel coronavirus" or "SARS-CoV" or "SARS-CoV-2" or "SARS2" or "2019-nCoV" or ncov19 or ncov-19 or "2019-novel CoV" or sarscov2 or sarscov-2 or Sars-coronavirus2 or Sars-coronavirus-2 or SARS-like coronavirus* or coronavirus-19).ti,ab,kf.
3	(coronavirus* or "corona virus*").ti,kf.
4	Coronavirus Infections/
5	or/1-4
6	20191201:20301231.(dt).
7	5 and 6 [COVID-19, SARS-COV-2]
8	limit 7 to English language
9	exp Neoplasms/
10	(cancer* or neoplas* or tumo?r* or leuk?emia* or lymphoma* or melanoma* or carcinoma* or sarcoma* or oncolog*).ti,ab,kf.
11	9 or 10 [Cancer]
12	8 and 11
13	Bone Marrow Transplantation/
14	exp Stem Cell Transplantation/
15	(("stem cell" or "bone marrow") adj3 transplant*).ti,ab,kf.
16	or/13-15 [Stem cell/bone marrow transplantation]
17	12 and 16 [COVID-19 + cancer patients + Stem cell/bone marrow transplantation]

Supplementary Table S2. Embase search strategy.

Search number	Search terms
1	("COVID-19" or COVID19 or "COVID 2019" or "novel coronavirus" or "SARS-CoV" or "SARS-CoV-2" or "SARS2" or "2019-nCoV" or ncov19 or ncov-19 or "2019-novel CoV" or sarscov2 or sarscov-2 or Sars-coronavirus2 or Sars-coronavirus-2 or SARS-like coronavirus* or coronavirus-19).ti,ab,kw.
2	(coronavirus* or "corona virus*").ti.
3	severe acute respiratory syndrome/
4	coronavirus infection/
5	"coronavirus disease 2019"/
6	or/1-5
7	limit 6 to English language
8	exp malignant neoplasm/
9	(cancer* or neoplas* or tumo?r* or leuk?emia* or lymphoma* or melanoma* or carcinoma* or sarcoma* or oncolog*).ti,ab.
10	8 or 9
11	7 and 10
12	exp bone marrow transplantation/
13	exp stem cell transplantation/
14	(("stem cell" or "bone marrow") adj3 transplant*).ti,ab.
15	or/12-14
16	11 and 15 [COVID-19 + cancer patients + Stem cell/bone marrow transplantation]

Supplementary Table S3. Web of Science search strategy.

Search number	Search terms
1	TOPIC: ((("COVID-19" or COVID19 or "COVID 2019" or "novel coronavirus" or "SARS-CoV" or "SARS-CoV-2" or "SARS2" or "2019-nCoV" or ncov19 or ncov-19 or "2019-novel CoV" or sarscov2 or sarscov-2 or Sars-coronavirus2 or Sars-coronavirus-2 or SARS-like coronavirus* or coronavirus-19)))
2	TITLE: ((coronavirus* or "corona virus*"))
3	#1 OR #2
4	TS=((cancer OR neoplasm OR tumor OR leukemia OR lymphoma OR melanoma OR carcinoma OR sarcoma OR oncolog*))
5	#4 AND #3
6	TOPIC: ((("stem cell" or "bone marrow") NEAR/3 transplant*))
7	#6 AND #5 Indexes=SCI-EXPANDED, SSCI, A&HCI, CPCI-S, CPCI-SSH, ESCI

Supplementary Table S4. Cochrane Library search strategy.

Search number	Search terms
1	MeSH descriptor: [Coronavirus Infections] explode all trees
2	("COVID-19" or COVID19 or "COVID 2019" or "novel coronavirus" or "SARS-CoV" or "SARS-CoV-2" or "SARS2" or "2019-nCoV" or ncov19 or ncov-19 or "2019-novel CoV" or sarscov2 or sarscov-2 or Sars-coronavirus2 or Sars-coronavirus-2 or "SARS-like coronavirus" or coronavirus-19):ti,ab,kw
3	(coronavirus or "corona virus"):ti
4	(coronavirus or "corona virus"):kw
5	(7-#4)
6	MeSH descriptor: [Neoplasms] explode all trees
7	((cancer or neoplas or tumour or leukemia or leukaemia or lymphoma or melanoma or carcinoma or sarcoma or oncolog* or malignanc*)):ti,ab,kw (Word variations have been searched)
8	#6 OR #7
9	#5 AND #8
10	MeSH descriptor: [Bone Marrow Transplantation] explode all trees
11	MeSH descriptor: [Cord Blood Stem Cell Transplantation] explode all trees
12	(("stem cell" or "bone marrow") NEAR/3 transplant*):ti,ab,kw
13	(25-#12)
14	#9 AND #13

Supplementary Table S5. Appraisal and quality assessment of the included studies

A. Case report appraisal

**Author/year**	Total Points
**Nazon 2020**	6/8
**Rossof 2020**	6/8
**De Giorgi 2020**	6/8
**Onaka 2020**	7/8
**Jarmolinski 2020**	7/8
**Flores 2020**	7/8
**Innes 2020**	7/8
**Karatas 2020**	7/8
**Angelini 2020**	7/8
**Nawar 2020**	7/8
**Huang 2020**	7/8
**Al Yazidi 2020**	7/8
**Balashov 2021**	7/8
**Foss 2020**	7/8
**Issa 2020**	7/8
**Krengli 2020**	7/8
**Lazaro Del Campo 2021**	6/8

B. Case series appraisal

**Author/year**	**Total points**
**Dufour 2020**	10/10
**Vicent 2020**	9/10
**Dhakal 2020**	9/10
**Haroon 2020**	9/10
**de Rojas 2020**	8/10
**Garnica 2020**	10/10
**Kanellopoulos 2020**	9/10
**Aydillo T 2020**	6/10
**Faura 2020**	9/10
**Niu 2020**	9/10

C. NOS **a**ppraisal

**Author/year**	Selection	Comparability	Outcome	Total
**Bisogno 2020**	3	1	2	6
**Pinana 2020**	4	2	3	9
**Shah 2020**	4	2	3	9
**Wang 2020**	4	2	3	9
**Fox 2020**	4	2	3	9
**Garcia-Suarez 2020**	4	2	3	9
**Jimenez-Kurlander 2021**	4	1	3	8
**Rouger-Gaudichon 2020**	3	1	3	7
**Sanchez-Pina 2020**	4	2	3	9

Supplementary Figure S1. Flow diagram of the Preferred Reporting Items for Systematic Reviews and Meta-Analyses guidelines.

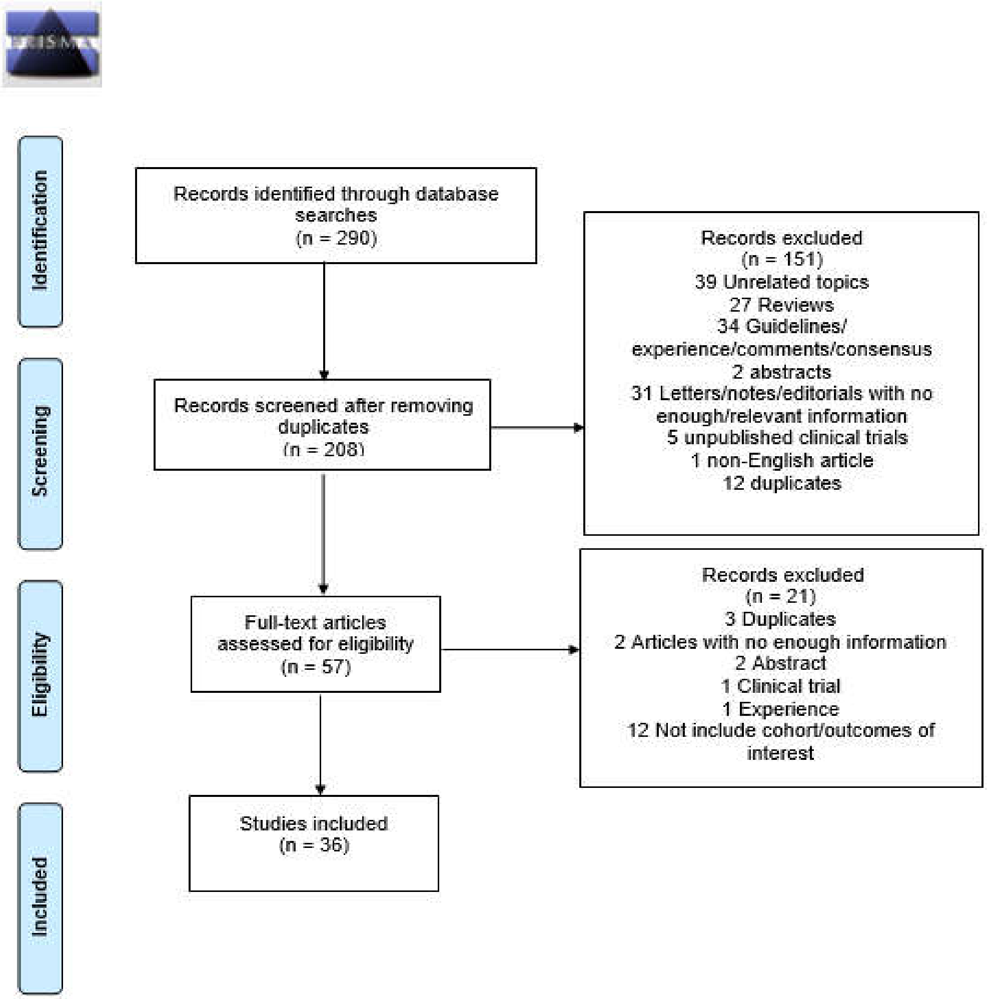

From: Moher D, Liberati A, Tetzlaff J, Altman DG, The PRISMA Group (2009). *Preferred Reporting Items for Systematic Reviews and Meta-Analyses*: The PRISMA Statement. PLoS Med 6(7): e1000097. doi:10.1371/journal.pmed1000097

For more information, visit www.prisma-statement.org.

Supplementary Figure S2. PERs for the use of antibiotics for treatment of COVID-19.

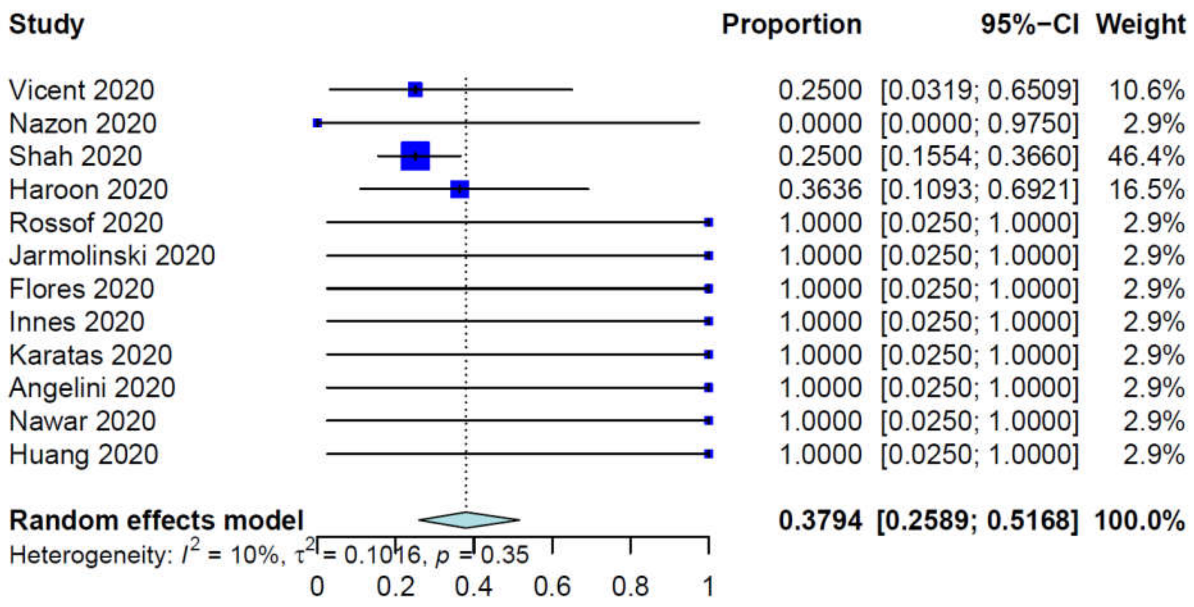

Supplementary Figure S3. PERs for the use of steroids for treatment of COVID-19.

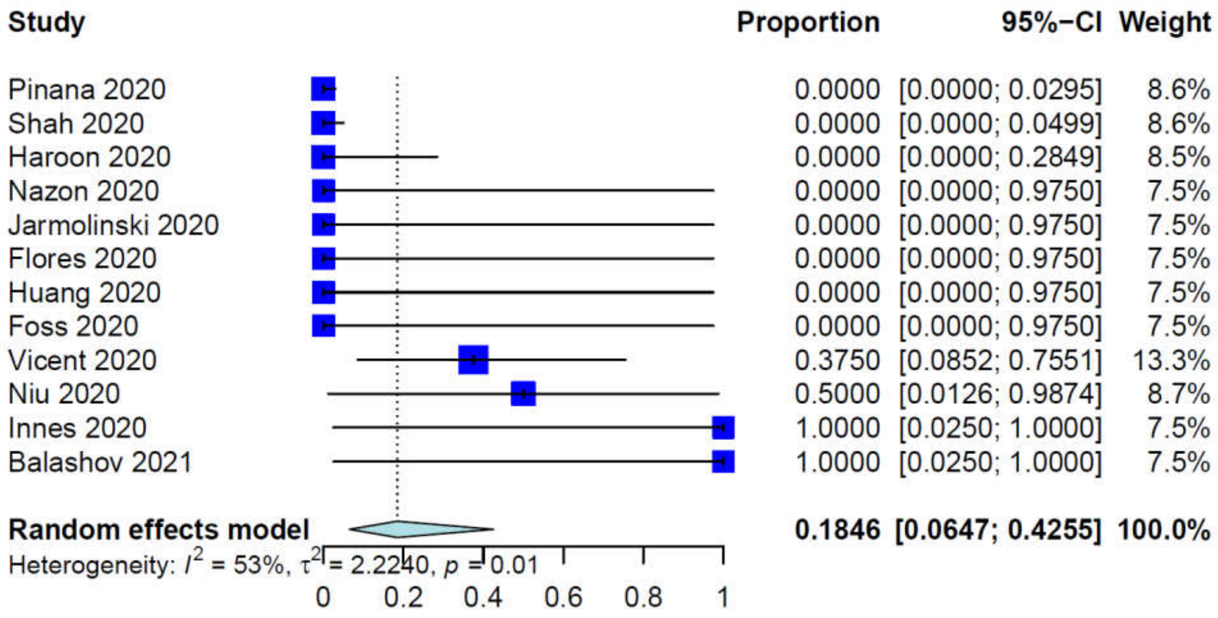

Supplementary Figure S4. PERs for the timing of SARS-CoV-2 infection in relation to SCT. (**A**) Zero to 100 days after SCT. (**B**) One hundred days to 6 months after SCT. (**C**) More than 6 months after SCT.

A

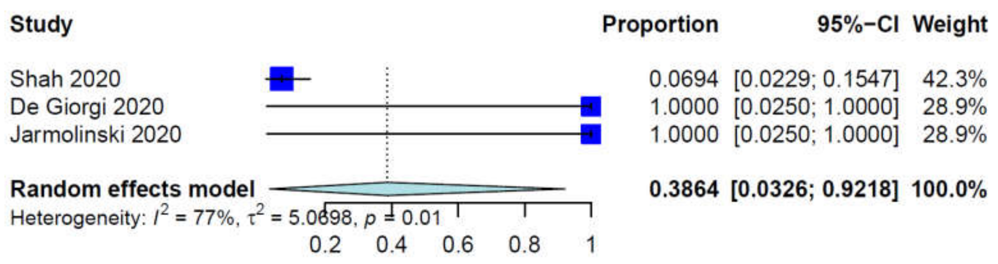

B

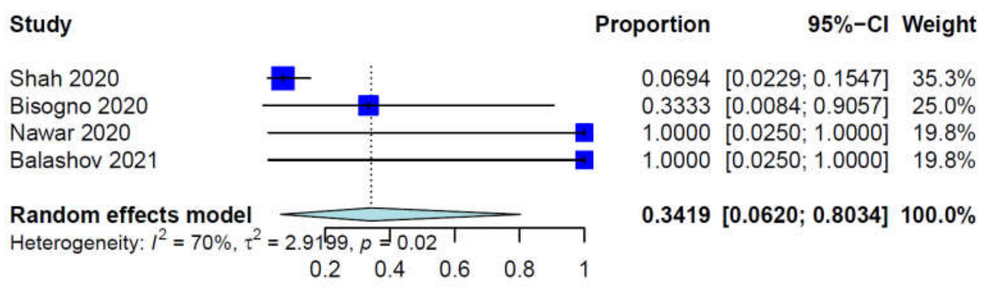

C

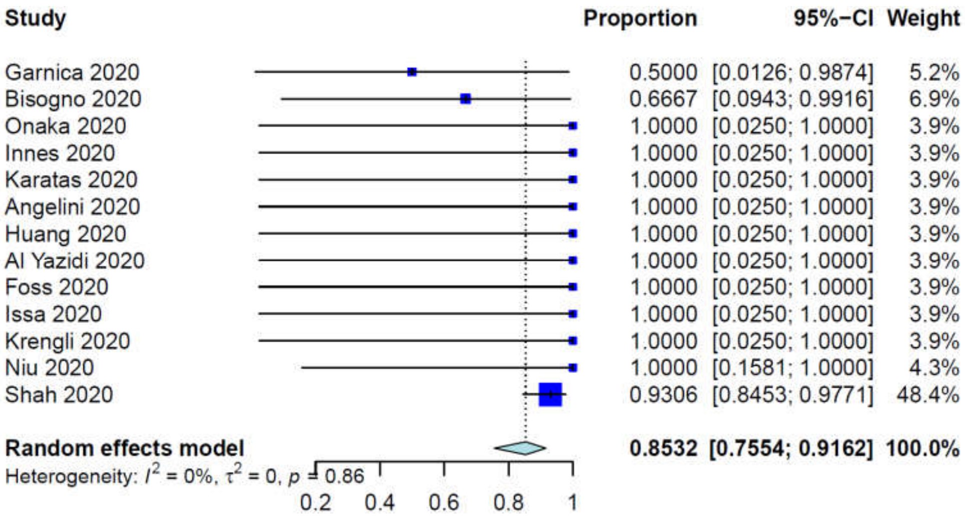
Study
Proportion
95%−CI
Weight
Garnica 2020 0.5000 [0.0126; 0.9874] 5.2%
Bisogno 2020 0.6667 [0.0943; 0.9916] 6.9%
Onaka 2020 1.0000 [0.0250; 1.0000] 3.9%
Innes 2020 1.0000 [0.0250; 1.0000] 3.9%
Karatas 2020 1.0000 [0.0250; 1.0000] 3.9%
Angelini 2020 1.0000 [0.0250; 1.0000] 3.9%
Huang 2020 1.0000 [0.0250; 1.0000] 3.9%
Al Yazidi 2020 1.0000 [0.0250; 1.0000] 3.9%
Foss 2020 1.0000 [0.0250; 1.0000] 3.9%
Issa 2020 1.0000 [0.0250; 1.0000] 3.9%
Krengli 2020 1.0000 [0.0250; 1.0000] 3.9%
Niu 2020 1.0000 [0.1581; 1.0000] 4.3%
Shah 2020 0.9306 [0.8453; 0.9771] 48.4%
Random effects model 0.8532 [0.7554; 0.9162] 100.0%
Heterogeneity: I^2 = 0%, τ^2 = 0, p = 0.86
0.2
0.4
0.6
0.8
1
